# Development of a Risk Prediction Model for Arteriovenous Fistula Restenosis Following Percutaneous Transluminal Angioplasty in Maintenance Hemodialysis Patients Using SMOTE Algorithm

**DOI:** 10.1155/cdr/5807867

**Published:** 2026-10-14

**Authors:** Jianping Yang, Yamei Zhu, Xiaoqing Zhu, Zhuangzhuang Guo, Mi Wang, Chensi Dong, Xueqiang Xu, Xianrong Xu

**Affiliations:** ^1^ Department of Nephrology Jiangsu Provincial People′s Hospital (The First Affiliated Hospital of Nanjing Medical University Jiangsu Provincial Maternity and Child Health Care Hospital) Nanjing Jiangsu China

**Keywords:** arteriovenous fistula, hemodialysis, machine learning, maintenance, nursing, percutaneous transluminal angioplasty, restenosis

## Abstract

**Background:**

Arteriovenous fistula (AVF) restenosis after percutaneous transluminal angioplasty (PTA) affects hemodialysis in maintenance hemodialysis (MHD) patients. Data imbalance limits predictive model accuracy.

**Objective:**

To identify risk factors and develop a SMOTE‐based risk prediction model for AVF restenosis within 12 months after PTA.

**Methods:**

This retrospective cohort study enrolled 241 consecutive MHD patients with AVF stenosis undergoing PTA between July 2022 and July 2025. Each patient contributed one index PTA. Patients were divided into restenosis (45 cases) and nonrestenosis (196 cases) groups according to whether AVF restenosis occurred within 12 months after PTA. Univariate and multivariate logistic regression identified risk factors. The SMOTE algorithm addressed data imbalance. Model performance was evaluated by ROC curves, calibration curves, and decision curve analysis (DCA).

**Results:**

Original‐data multivariate analysis identified multisegmental stenosis, cannulation method, platelet count, and C‐reactive protein (CRP) as independent risk factors. After SMOTE, intradialytic hypotension and serum albumin also became significant; however, these variables should be interpreted as exploratory because SMOTE does not create additional independent clinical information. The SMOTE‐based model (P2) achieved AUC of 0.934 (training set) and 0.868 (validation set), outperforming the original model (P1).

**Conclusion:**

The SMOTE‐based model may help predict AVF restenosis within 12 months after PTA, but it is an exploratory internally validated tool and requires external validation before clinical use.

## 1. Introduction

Percutaneous transluminal angioplasty (PTA) currently serves as one of the primary interventions for stenosis in autologous arteriovenous fistula (AVF). Compared with surgical fistula revision, PTA is minimally invasive, requires no significant incision, is relatively straightforward to perform, and can be repeated multiple times. The dilated stenotic vessel can be immediately reused, with a success rate ranging from 82% to 94% [1]. This approach significantly preserves the limited vascular resources in patients undergoing maintenance hemodialysis (MHD) [2]. However, because of underlying patient comorbidities, physicochemical factors, intrinsic vascular structure, and mechanical dilation by balloons during the procedure, vascular endothelial cells are inevitably injured, leading to stimulation of vascular smooth muscle and subsequent activation and proliferation of macrophages. These processes contribute to AVF restenosis [3], often necessitating two or more PTA sessions. This increases the technical difficulty of subsequent treatments, reduces long‐term patency rates, consumes considerable healthcare resources, and imposes substantial economic burdens and physical distress on MHD patients [4]. Therefore, investigating risk factors for AVF restenosis after PTA and implementing timely and effective interventions for such patients are critical to preventing its occurrence. However, studies often face data imbalance between restenosis and nonrestenosis cases following PTA. The synthetic minority oversampling technique (SMOTE), a resampling method that balances minority and majority class data [5], has become a widely adopted approach for addressing imbalanced datasets. This study is aimed at developing a predictive model for AVF restenosis after PTA based on the SMOTE algorithm, with the goal of identifying high‐risk factors and providing valuable insights to reduce the incidence of clinical AVF restenosis post‐PTA.

## 2. Objects and Methods

### 2.1. Study Population

A total of 241 MHD patients who underwent PTA for AVF stenosis at the Department of Nephrology, Jiangsu Provincial People′s Hospital between July 2022 and July 2025 were enrolled. All cases involved lower‐arm AVFs with documented stenosis ranging from mild, moderate, severe, to critical; some presented with multiple stenotic lesions. Stenotic vessel length varied from 5 to 80 mm, with the narrowest luminal diameter measuring 0.3–3.1 mm. All patients underwent PTA for AVF stenosis, and relevant clinical data were collected. Each patient contributed only one index PTA. Patients with previous PTA within 3 months were excluded. All patients were followed for at least 12 months after PTA or until restenosis occurred. The nonrestenosis group was defined as patients without AVF restenosis during the 12‐month follow‐up.

Inclusion criteria: (1) hospitalization because of AVF stenosis with indications for endovascular intervention; diagnostic criteria for native AVF stenosis included: (a) color Doppler ultrasonography showing ≥ 50% luminal narrowing compared with the adjacent normal vessel, accompanied by one or more of the following: (i) native AVF blood flow < 500 mL/min; (ii) pump‐controlled or access blood flow reduction < 200 mL/min; (iii) abnormal physical signs of AVF; (iv) cannulation difficulty; (v) significant elevation of venous pressure during dialysis; (vi) reduced dialysis adequacy [6]; (b) asymptomatic with stenosis > 70%; (2) age ≥ 18 years; (3) regular hemodialysis for ≥ 3 months; (4) provision of informed consent by the patient or legal guardian; (5) complete and accurate clinical documentation.

Exclusion criteria were: (1) AVF occlusion, thrombosis, infection, or severe coagulopathy; (2) stenosis combined with pseudoaneurysm; (3) concomitant ipsilateral central venous stenosis or occlusion; (4) history of psychiatric disorders, malignancy, or autoimmune diseases; (5) severe cardiac, pulmonary, hepatic, or other organ dysfunction; (6) cognitive impairment precluding study compliance. The study protocol was approved by the Institutional Ethics Committee (Approval No. 2023‐SR‐136).

### 2.2. Methods

#### 2.2.1. Grouping and Data Collection

Patients were stratified into restenosis and nonrestenosis groups based on AVF restenosis within 12 months after PTA. Restenosis was defined as recurrent AVF stenosis requiring repeat PTA or surgical revision, confirmed by color Doppler ultrasonography showing ≥ 50% luminal narrowing compared with the adjacent normal vessel, or by clinical criteria including access flow reduction, cannulation difficulty, elevated venous pressure, or reduced dialysis adequacy. Clinical parameters collected included sex, age, primary disease, comorbidities (hypertension, diabetes), intradialytic hypotension (defined as systolic blood pressure drop ≥ 20 mmHg or mean arterial pressure drop ≥ 10 mmHg, accompanied by symptoms such as nausea, dyspnea, restlessness, or drowsiness) [7], dialysis vintage, current AVF age, and pre‐PTA color Doppler parameters (measured by dedicated vascular access ultrasonographers). Parameters included AVF flow rate, anastomosis size, arterial diameter, presence of multisegment stenosis, stenotic segment length, minimal luminal diameter, cannulation method, and laboratory results 1–2 days pre‐PTA: hemoglobin, platelet count, D‐dimer, C‐reactive protein, total cholesterol, triglycerides, high‐density lipoprotein cholesterol (HDL‐C), low‐density lipoprotein cholesterol (LDL‐C), blood urea nitrogen, serum creatinine, calcium, phosphate, and calcium‐phosphate product.

#### 2.2.2. SMOTE Oversampling Algorithm

The SMOTE was employed to augment the minority class (restenosis group, *n* = 30) in the training set [8]. The expansion factor was calculated as the ratio of the majority to minority class in the training set (131/30 ≈ 4.37). Using *k* = 4 nearest neighbors, 90 synthetic samples were generated, resulting in a total of 120 restenosis cases (30 original + 90 synthetic) and 131 nonrestenosis cases, with a ratio of 1.09:1. This approach preserves the original data distribution and ensures algorithmic reliability.

### 2.3. Statistical Methodology

Data from both groups were processed and analyzed using SPSS 27. Categorical data were expressed as counts and percentages and analyzed using the chi‐square (*χ* ^2^) test. Continuous data were presented as mean ± standard deviation ($\bar{x}\pm s$) and assessed with the *t*‐test. Risk factors associated with restenosis of AVF following PTA in MHD patients were screened using logistic regression analysis. Predictive modeling was conducted using R software (Version 4.2.1), with the construction of nomograms, receiver operating characteristic (ROC) curves, and calibration curves. The discriminative ability of the model was evaluated via decision curve analysis (DCA), whereas the Hosmer–Lemeshow test was employed to assess model calibration. Model accuracy was validated using the F‐score, true positive rate (TPR), and positive predictive value (PPV). A two‐sided *p* − value < 0.05 was considered statistically significant.

## 3. Results

### 3.1. Restenosis Risk and Dataset Partition of Enrolled Patients

This study included 241 patients who underwent PTA for stenotic AVF. Among them, 45 cases developed restenosis, whereas 196 cases did not, yielding a restenosis incidence of 18.67% (45/241). The enrolled patients were randomly allocated in a 2:1 ratio into a training set (*n* = 161) and a validation set (*n* = 80). The training set included 30 restenosis and 131 nonrestenosis cases. The validation set included 15 restenosis and 65 nonrestenosis cases. No statistically significant differences were observed in any baseline characteristics between the two groups, indicating comparability (*p* > 0.05) (Table 1). Among the 45 patients with restenosis, the mean time to restenosis was 6.2 ± 3.1 months (range: 1.5–12.0 months). No cases of technical failure (restenosis within 30 days post‐PTA) were observed in this study, confirming that all events were true biological restenosis rather than procedural failure.

**Table 1:** Comparison of baseline clinical characteristics between training and validation sets.

Project	Training set (*n* = 161)	Validation set (*n* = 80)	*t*/*χ* ^2^ values	*p*
Gender [*n* (%)]			0.002	0.969
Male	93 (57.8)	46 (57.5)		
Female	68 (42.2)	34 (42.5)		
Age [*y*, ($\bar{x}\pm s$)]	58.61 ± 13.05	60.90 ± 12.66	−1.296	0.196
Primary disease [*n* (%)]			0.668	0.881
Chronic glomerulonephritis	140 (87.0)	71 (88.7)		
Diabetic nephropathy	9 (5.5)	5 (6.3)		
Polycystic kidney	8 (5.0)	3 (3.7)		
Others	4 (2.5)	1 (1.3)		
Combined hypertension [*n* (%)]			0.084	0.772
Yes	141 (87.6)	69 (86.3)		
No	20 (12.4)	11 (13.8)		
Combined diabetes [*n* (%)]			0.168	0.682
Yes	78 (48.4)	41 (51.2)		
No	83 (51.6)	39 (48.8)		
Combined coronary heart disease			0.007	0.933
Yes	37 (23.0)	18 (22.5)		
No	124 (77.0)	62 (77.5)		
In dialysis hypotension [*n* (%)]			0.863	0.353
Have	14 (8.7)	10 (12.5)		
None	147 (91.3)	70 (87.5)		
Duration of dialysis [*y*, ($\bar{x}\pm s$)]	4.32 ± 5.12	4.38 ± 4.98	−0.084	0.933
Duration of current arteriovenous fistula [*y*, (^−^ *x* ± *s*)]	3.36 ± 4.46	3.70 ± 4.73	−0.541	0.589
AVF flow rate [mL/min, ($\bar{x}\pm s$)]	343.58 ± 98.53	351.21 ± 170.76	−0.439	0.661
Anastomosis diameter [mm, ($\bar{x}\pm s$)]	9.21 ± 3.38	8.45 ± 4.32	1.491	0.137
Fistula brachial artery diameter [mm, ($\bar{x}\pm s$)]	2.43 ± 1.36	2.35 ± 0.95	0.448	0.654
Multi‐segment stenosis [*n* (%)]			0.483	0.487
Yes	45 (28.0)	19 (23.8)		
No	116 (72.0)	61 (76.2)		
Stenosis length > 2 cm [*n* (%)]			1.145	0.285
Yes	57 (35.4)	34 (42.5)		
No	104 (64.6)	46 (57.5)		
Minimum luminal diameter at the narrowest segment [mm, ($\bar{x}\pm s$)]	0.79 ± 0.40	0.72 ± 0.36	1.406	0.161
Puncture method [*n* (%)]			1.613	0.204
Buttonhole cannulation	13 (8.1)	3 (3.8)		
Rope‐ladder cannulation	148 (91.9)	77 (96.2)		
Hemoglobin [g/L, ($\bar{x}\pm s$)]	106.38 ± 20.13	109.74 ± 19.86	−1.225	0.222
The platelet count [x 109/L, ($\bar{x}\pm s$)]	172.50 ± 62.15	160.63 ± 57.99	1.427	0.155
D‐dimer [*μ*g/mL, ($\bar{x}\pm s$)]	0.98 ± 1.02	1.12 ± 1.67	−0.797	0.426
C‐reactive protein (mg/L, ($\bar{x}\pm s$)]	5.44 ± 7.57	7.09 ± 9.94	−1.427	0.155
Serum albumin [g/L, ($\bar{x}\pm s$)]	36.47 ± 4.29	36.01 ± 5.46	0.708	0.480
Cholesterol [mmol/L, ($\bar{x}\pm s$)]	3.67 ± 1.26	3.66 ± 1.19	0.062	0.951
Triglycerides [mmol/L, ($\bar{x}\pm s$)]	1.76 ± 1.20	1.58 ± 0.89	1.199	0.232
HDL‐C[mmol/L, ($\bar{x}\pm s$)]	0.95 ± 0.33	0.94 ± 0.24	0.282	0.778
LDL‐C[mmol/L, ($\bar{x}\pm s$)]	2.25 ± 0.88	2.23 ± 0.82	0.179	0.858
Blood urea [mmol/L, ($\bar{x}\pm s$)]	25.73 ± 41.87	21.83 ± 6.86	0.826	0.409
Serum creatinine [*μ*mol/L, ($\bar{x}\pm s$)]	755.27 ± 296.38	719.59 ± 262.43	0.913	0.362
Serum calcium [mmol/L, ($\bar{x}\pm s$)]	2.22 ± 0.22	2.25 ± 0.23	−0.869	0.386
Blood phosphorus [mmol/L, ($\bar{x}\pm s$)]	1.75 ± 0.50	1.89 ± 0.58	−1.974	0.051
Calcium‐phosphorus product [mg/dL, ($\bar{x}\pm s$)]	48.51 ± 15.44	51.43 ± 15.14	−1.392	0.165

### 3.2. Univariate Analysis of AVF Restenosis After PTA in MHD Patients

Based on the occurrence of restenosis, patients in the training set were divided into the restenosis group (*n* = 30) and the nonrestenosis group (*n* = 131). No statistically significant differences were observed between the two groups regarding gender, age, primary disease, hypertension, diabetes, coronary heart disease, dialysis duration, current AVF duration, anastomotic orifice size, brachial artery internal diameter at the fistula site, hemoglobin, D‐dimer, cholesterol, triglycerides, HDL‐C, LDL‐C, blood urea nitrogen, serum creatinine, and serum calcium levels (*p* > 0.05). However, statistically significant differences (*p* < 0.05) were identified in the proportion of intradialytic hypotension, fistula flow rate, presence of multiple stenosis segments in the fistula, proportion of stenosis length > 2 cm, minimum internal diameter at the most stenotic site, puncture method, platelet count, C‐reactive protein, serum albumin, serum phosphorus, and calcium‐phosphorus product (Table 2).

**Table 2:** Comparison of baseline clinical characteristics and univariate analysis of restenosis between the two patient groups.

Project	Restenosis group (*n* = 30)	Nonrestenosis group (*n* = 131)	*t*/*χ* ^2^ values	*p*
Gender [*n* (%)]			0.469	0.494
Male	19 (63.3)	74 (56.5)		
Female	11 (36.7)	57 (43.5)		
Age [*n* (%)]			0.448	0.503
≥ 60	16 (53.3)	61 (46.6)		
The < 60	14 (46.7)	70 (53.4)		
The primary disease [*n* (%)]			2.267	0.519
Chronic glomerulonephritis	24 (80.0)	116 (88.5)		
Diabetic kidney disease	2 (6.7)	7 (5.3)		
Polycystic kidney	3 (10.0)	5 (3.9)		
Others	1 (3.3)	3 (2.3)		
Hypertension [*n* (%)]			1.123	0.289
Yes	28 (93.3)	113 (86.3)		
No	2 (6.7)	18 (13.7)		
Combined diabetes [*n* (%)]			0.36	0.85
Yes	15 (50.0)	63 (48.1)		
No	15 (50.0)	68 (51.9)		
Combined coronary heart disease			0.283	0.595
Yes	8 (26.7)	29 (22.1)		
No	22 (73.3)	102 (77.9)		
In dialysis hypotension [*n* (%)]			5.934	0.015
Have	6 (20.0)	8 (6.1)		
None	24 (80.0)	123 (93.9)		
[dialysis duration *y*, $\left(\bar{x}\pm s\right)$]	5.10 ± 4.7	4.08 ± 5.03	1.017	0.311
Duration of current arteriovenous fistula [*y*, ($\bar{x}\pm s$)]	3.93 ± 4.06	3.23 ± 4.55	0.776	0.439
AVF flow rate [mL/min, ($\bar{x}\pm s$)]	294.90 ± 115.75	354.73 ± 91.04	−2.649	0.012
Anastomosis diameter [mm, ($\bar{x}\pm s$)]	9.39 ± 2.82	9.17 ± 3.51	0.328	0.743
Fistula brachial artery diameter [mm, ($\bar{x}\pm s$)]	2.53 ± 1.48	2.41 ± 1.33	0.453	0.651
Multisegmental stenosis [*n* (%)]			49.602	< 0.001
Yes	24 (80.0)	21 (16.0)		
No	6 (20.0)	110 (84.0)		
Stenosis length > 2 cm [*n* (%)]			15.763	< 0.001
Yes	20 (66.7)	37 (28.2)		
No	10 (33.3)	94 (71.8)		
The narrow place inside diameter (mm, ($\bar{x}\pm s$)]	0.69 ± 0.24	0.82 ± 0.42	−2.179	0.032
Puncture method [*n* (%)]			23.879	< 0.001
Buttonhole cannulation	9 (30.0)	4 (3.1)		
Ladder type puncture method	21 (70.0)	127 (96.9)		
Hemoglobin (g/L, ($\bar{x}\pm s$)]	105.47 ± 18.66	106.59 ± 20.51	−0.274	0.784
Platelet count [×10^9^/L, ($\bar{x}\pm s$)]	199.63 ± 67.56	166.28 ± 59.39	2.703	0.008
D‐dimer [*μ*g/mL, ($\bar{x}\pm s$)]	0.72 ± 0.67	1.04 ± 1.08	−1.559	0.121
C‐reactive protein (mg/L, ($\bar{x}\pm s$)]	8.31 ± 7.95	4.79 ± 7.36	2.331	0.021
Serum albumin [g/L, ($\bar{x}\pm s$)]	35.42 ± 2.71	36.71 ± 4.55	−2.029	0.046
Cholesterol [mmol/L, ($\bar{x}\pm s$)]	3.73 ± 1.21	3.65 ± 1.28	0.281	0.779
Triglycerides [mmol/L, ($\bar{x}\pm s$)]	1.87 ± 1.43	1.73 ± 1.14	0.583	0.560
HDL‐C [mmol/, ($\bar{x}\pm s$)]	0.92 ± 0.34	0.96 ± 0.33	−0.543	0.588
LDL‐C [mmol/L, ($\bar{x}\pm s$)]	2.28 ± 0.85	2.25 ± 0.89	0.188	0.851
Blood urea mmol/L, ($\bar{x}\pm s$)]	38.95 ± 95.30	22.70 ± 9.06	0.933	0.359
Serum creatinine [*μ*mol/L, ($\bar{x}\pm s$)]	823.10 ± 298.55	739.73 ± 294.83	1.394	0.165
Blood calcium [mmol/L, ($\bar{x}\pm s$)]	2.23 ± 0.30	2.22 ± 0.20	0.105	0.916
Blood phosphorus [mmol/L, ($\bar{x}\pm s$)]	2.01 ± 0.63	1.69 ± 0.45	2.638	0.012
Calcium‐phosphorus product [mg/dL ($\bar{x}\pm s$)]	56.19 ± 19.51	46.76 ± 13.85	2.509	0.017

### 3.3. Multivariate Analysis of AVF Restenosis Following PTA in MHD Patients

Variables that demonstrated statistical significance in the preceding univariate analysis—including intradialytic hypotension proportion, fistula flow rate, proportion of multisegmental stenosis, proportion of stenosis length > 2 cm, minimum luminal diameter at the narrowest segment, proportion of cannulation methods, platelet count, C‐reactive protein, serum albumin, serum phosphorus, and calcium‐phosphorus product—were designated as independent variables. AVF restenosis after PTA was defined as the dependent variable, with 1 indicating restenosis and 0 indicating no restenosis. A binary logistic regression analysis was conducted to identify risk factors for AVF restenosis post‐PTA. Original‐data multivariate logistic regression showed that multisegmental stenosis (*β* = 2.954, OR = 19.183, 95% CI 4.148–88.884, *p* < 0.001), cannulation method (*β* = 3.296, OR = 27.013, 95% CI 3.725–195.904, *p* = 0.001), platelet count (*β* = 0.014, OR = 1.014, 95% CI 1.004–1.025, *p* = 0.007), and CRP (*β* = 0.096, OR = 1.101, 95% CI 1.030–1.176, *p* = 0.005) were independent risk factors for AVF restenosis. Intradialytic hypotension (*p* = 0.124) and serum albumin (*p* = 0.098) were not statistically significant in the original‐data model. Based on the risk factors and regression coefficients from the univariate analysis, the initial binary logistic regression model was derived as follows:

P10.0042.9540.4771.2873.296=1.579X1–X2+X3+X4–X5+X6+0.0140.0960.1272.2350.0173.362X7+X8–X9+X10–X11–.

The predicted probability was: *p* = 1/(1 + *e*∧(−*P*1)).

Variable definitions:

*X*1 = intradialytic hypotension (0 = no, 1 = yes).

*X*2 = AVF flow rate (mL/min, continuous).

*X*3 = multisegmental stenosis (0 = no, 1 = yes).

*X*4 = stenosis length > 2 cm (0 = no, 1 = yes).

*X*5 = minimum luminal diameter at the narrowest segment (mm, continuous).

*X*6 = cannulation method (0 = rope − ladder, 1 = buttonhole).

*X*7 = platelet count (×10^9/L, continuous).

*X*8 = CRP (mg/L, continuous)

*X*9 = serum albumin (g/L, continuous).

*X*10 = serum phosphorus (mmol/L, continuous).

*X*11 = calcium − phosphorus product (mg/dL, continuous).

The Hosmer–Lemeshow test indicated adequate model fit (coefficient of determination *R* ^2^ = 0.620, *p* = 0.256) (Table 3).

**Table 3:** Binary logistic regression analysis of AVF restenosis after PTA using original data.

Items	*β* values	SE values	Wald *x* ^2^ value	*p*	The OR value	95% CI	
						Lower limit	Upper limit
Hypotension during dialysis	1.579	1.027	2.364	0.124	4.848	0.648	36.270
Arteriovenous fistula flow	−0.004	0.004	1.147	0.284	0.996	0.989	1.003
Multisegment stenosis	2.179	0.491	19.695	< 0.001	8.837	3.376	23.135
Stenosis length >2 cm	0.498	0.415	1.440	0.203	1.645	0.729	3.711
The inner diameter at the narrowest point	−1.287	0.982	1.716	0.190	0.276	0.040	1.894
The puncture method	3.296	1.011	10.633	0.001	27.013	3.725	195.904
Platelet count	0.014	0.005	7.179	0.007	1.014	1.004	1.025
C‐reactive protein	0.096	0.034	8.041	0.005	1.101	1.030	1.176
Serum albumin	−0.127	0.077	2.730	0.098	0.880	0.757	1.024
Blood phosphorus	2.235	2.059	1.179	0.278	9.347	0.165	528.309
Calcium‐phosphorus product	−0.017	0.068	0.059	0.807	0.984	0.861	1.123
Constant	−3.362	3.579	0.882	0.348	0.035	—	—

*Note:* These estimates are based on observed clinical events only.

### 3.4. Construction of the SMOTE‐Logistic Model P2 Using the SMOTE Algorithm

A four‐fold oversampling was performed using the SMOTE algorithm, resulting in a ratio of 1.09 (approximated to 1) between the postoperative AVF restenosis and nonrestenosis groups. The SMOTE‐logistic regression model derived from the oversampling process is expressed as

P22.270.0023.1170.7261.4332.907=X1−X2+X3+X4−X5+X6+0.0170.1200.1261.8900.0133.002X7+X8−X9+X10−X11−.

The predicted probability was: *p* = 1/(1 + *e*∧(−*P*2)). Variable definitions are the same as above.

The Hosmer–Lemeshow test indicated a good model fit (coefficient of determination *R* ^2^ = 0.699, *p* = 0.220) (Table 4). After SMOTE, intradialytic hypotension (*p* = 0.003) and serum albumin (*p* = 0.020) became significant. However, SMOTE only generates synthetic samples and does not create new independent clinical information. Therefore, these two variables should be regarded as exploratory findings and should not be interpreted as independent risk factors equivalent to those identified in the original‐data model.

**Table 4:** Binary logistic regression analysis after SMOTE.

Items	*β* values	SE values	Wald *x* ^2^ value	*p*	The OR value	95% CI	
						Lower limit	Upper limit
Hypotension during dialysis	2.27	0.763	8.855	0.003	9.678	2.17	43.159
Arteriovenous fistula flow	−0.002	0.002	0.738	0.390	0.998	0.994	1.002
Multisegment stenosis	3.117	0.510	37.301	< 0.001	22.581	8.304	61.410
Narrow segments > 2 cm in length	0.726	0.483	2.259	0.133	2.066	0.802	5.322
The inner diameter at the narrowest point	−1.433	0.737	3.778	0.052	0.239	0.056	1.012
The puncture method	2.907	0.715	16.515	< 0.001	18.300	4.504	74.355
Platelet count	0.017	0.004	16.855	< 0.001	1.018	1.009	1.026
C‐reactive protein	0.120	0.030	15.746	< 0.001	1.127	1.063	1.196
Serum albumin	−0.126	0.054	5.411	0.020	0.882	0.793	0.980
Blood phosphorus	1.890	1.491	1.607	0.205	6.619	0.356	122.592
Calcium‐phosphorus product	−0.013	0.048	0.073	0.788	0.987	0.898	1.085
Constant	−3.002	2.436	1.519	0.218	0.050	—	—

*Note:* These estimates are based on a dataset containing synthetic minority samples and should be interpreted as exploratory. SMOTE does not create additional independent clinical information.

### 3.5. Development of a Nomogram Model for AVF Restenosis After PTA in MHD Patients and Performance Evaluation of Two Predictive Models

Eleven risk factors were incorporated into R software to construct a nomogram model for predicting AVF restenosis after PTA in MHD patients (Figure 1). To evaluate the predictive performance of the two regression models (P1 and P2), ROC curve analysis was conducted. The results demonstrated that in the training set, the AUC for model P1 was 0.931 (95% CI: 0.884–0.978), while that for model P2 was 0.934 (95% CI: 0.906–0.962). In the validation set, the AUC for model P1 was 0.844 (95% CI: 0.783–0.904), and for model P2, it was 0.868 (95% CI: 0.813–0.923) (Figure 2). The classification threshold was defined as the predicted probability at the maximum Youden index in the training set. For P1, the exact threshold was 0.436 (Youden index = 0.757); for P2, the exact threshold was 0.492 (Youden index = 0.764). The same thresholds were applied to the validation set (P1 validation: 0.436; P2 validation: 0.492). TPR, PPV, specificity, NPV, and F‐score in Table 5 were calculated at these fixed thresholds. In the validation cohort, the calibration curve of model P2 indicated strong agreement between predicted and observed outcomes (Figure 3). The DCA of the nomogram suggested that timely intervention in high‐risk MHD patients for AVF restenosis after PTA would benefit the majority of patients, underscoring the model′s considerable clinical utility (Figure 4).

**Figure 1:**
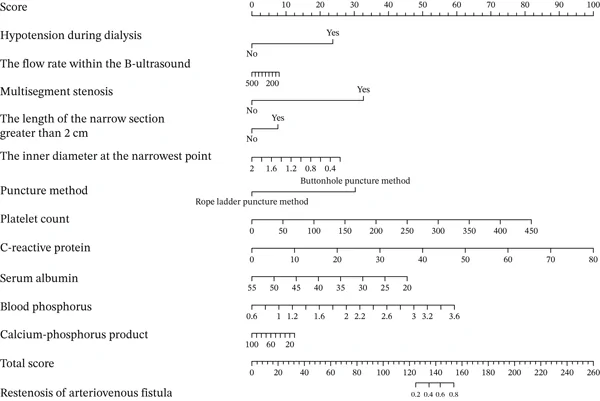
Nomogram for predicting the risk of AVF restenosis after PTA in MHD patients.

**Figure 2:**
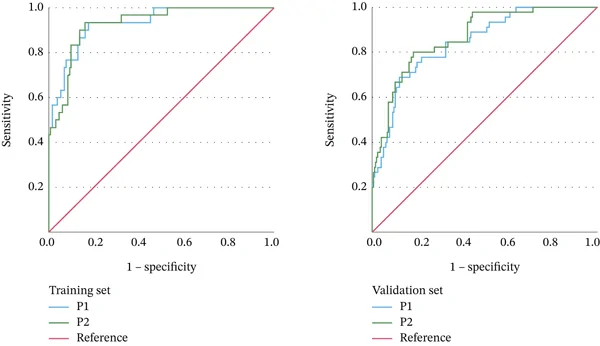
ROC curves of the original logistic model (P1) versus the SMOTE‐logistic model (P2).

**Table 5:** Calibration validation of probabilities between the original predictive model (P1) and the SMOTE‐based predictive model (P2).

Model	Dataset	AUC	95% CI	Threshold	TPR	F‐score	PPV	Specificity	NPV
P1	Training set	0.931	0.884, 0.978	0.436	0.933	0.690	0.548	0.824	0.982
P1	Validation set	0.844	0.783, 0.904	0.436	0.845	0.678	0.535	0.789	0.965
P2	Training set	0.934	0.906, 0.962	0.492	0.901	0.746	0.622	0.863	0.892
P2	Validation set	0.868	0.813, 0.923	0.492	0.863	0.721	0.612	0.852	0.881

*Note:* Thresholds were determined in the training set by the maximum Youden index and then fixed for the validation set: P1 = 0.436; P2 = 0.492.

**Figure 3:**
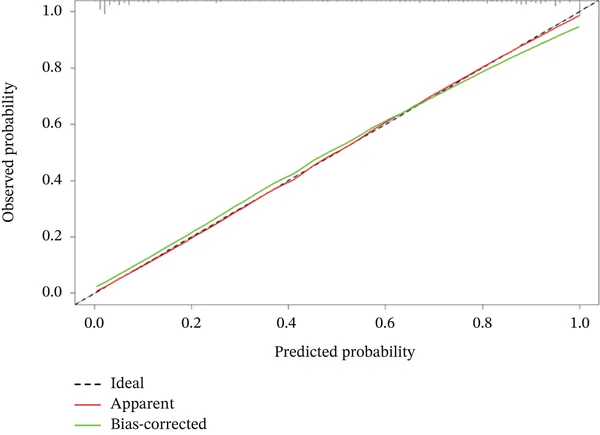
Calibration curve of the SMOTE‐based predictive model for AVF restenosis risk after PTA in MHD patients.

**Figure 4:**
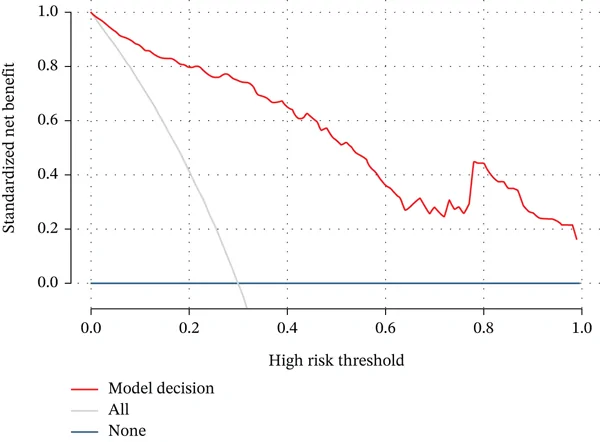
Clinical decision curve analysis of the nomogram.

## 4. Discussion

Vascular stenosis represents the most common long‐term complication of vascular access in MHD patients and is one of the principal causes of AVF failure. After PTA, stenotic vessels can be immediately reused with favorable clinical outcomes. However, according to Zhan et al. [9], the primary patency rates at 6‐months and 12‐months post‐PTA were 77.31% and 50.15%, respectively, indicating that some patients still experience AVF restenosis after PTA. In our center, the incidence of AVF restenosis was 18.63%.

The present study revealed that intradialytic hypotension is a risk factor for AVF restenosis after PTA in MHD patients, which aligns with the findings of Gong et al. [10]. During hypotensive episodes, decreased vascular wall tension leads to a significant reduction in AVF blood flow, increasing the risk of thrombosis and subsequent stenosis or occlusion of the AVF. Therefore, during hypotension in MHD patients, ultrafiltration should be promptly reduced or discontinued; after stabilization of vital signs, ultrafiltration should be resumed gradually. Dry weight should be assessed in a timely manner, and the dosage and administration timing of antihypertensive medications should be adjusted to reduce the incidence of intradialytic hypotension and prevent AVF restenosis. Additionally, our results indicate that the presence of multisegment stenosis at the initial intervention is a risk factor for AVF restenosis post‐PTA. Most patients presented with stenoses at more than one site, leading to increased cumulative lesion length. In this study, a lesion length > 20 mm was identified as a significant predictor in univariate analysis (*p* < 0.05). Clark et al. [11] reported that the risk of AVF failure in patients with lesion lengths > 20 mm was five times higher than in those with lesions < 20 mm. Another study by Zhan et al. [12] determined a cutoff cumulative lesion length of 36 mm, with significantly higher 12‐month primary patency rates in patients with lesions < 36 mm compared with those ≥ 36 mm. Thus, early PTA intervention is recommended upon detection of AVF stenosis to prevent the progression to multisegment involvement and increased cumulative lesion length.

Moreover, the buttonhole cannulation technique was identified as a risk factor for AVF restenosis following PTA. Zhao et al. [13] observed vascular intimal hyperplasia of varying severity in 68.2% of patients using this method. Local inflammatory responses induced by buttonhole cannulation also contribute to intimal thickening, thereby promoting restenosis. Therefore, the rope‐ladder cannulation technique is recommended, accompanied by routine vascular ultrasonography every 3 months to enable early detection of complications such as stenosis or thrombosis. Elevated platelet count was also associated with an increased risk of AVF restenosis after PTA. Platelet count is a sensitive indicator of blood viscosity; higher levels increase blood viscosity and slow velocity, accelerating thrombus formation—a result consistent with the study by Weng et al. [14]. Higher platelet counts correlate with an increased risk of late AVF failure. This study suggests that appropriate anticoagulants or antiplatelet agents should be administered during dialysis after PTA to prevent thrombosis through early prophylactic intervention.

Furthermore, elevated C‐reactive protein levels were associated with a higher risk of AVF restenosis post‐PTA in MHD patients, corroborating previous findings by Wang et al. [15].. Increased C‐reactive protein indicates activation of inflammatory pathways, which stimulates the complement and coagulation systems, leading to an imbalance in fibrinolysis, thrombosis, and potential disseminated intravascular coagulation. Thus, strict aseptic techniques during AVF cannulation and needle removal, as well as during dialysis circuit setup and drug administration, are essential to reduce C‐reactive protein levels, prevent recurrent thrombosis, and minimize restenosis risk. Finally, decreased serum albumin levels were found to increase the risk of AVF restoration after PTA in MHD patients, consistent with Zhang et al. [16]. Low serum albumin levels exacerbate vascular endothelial injury, activate the coagulation system, increase blood viscosity, and promote intravascular thrombosis, thereby impairing nutrient delivery and contributing to AVF dysfunction. Therefore, patient education on high‐quality, high‐protein diets is crucial to maintain adequate serum albumin levels and reduce the risk of AVF restenosis post‐PTA.

This study training set involved 161 MHD patients who underwent PTA. Postoperative AVF restenosis occurred in 18.63% (30/161) of cases, whereas 81.37% (131/161) did not develop restenosis. The marked imbalance between the two groups may adversely affect the predictive accuracy of the model. To address this clinical data imbalance, the SMOTE algorithm was applied to generate a new sample set in which the ratio of restenosis to nonrestenosis groups approximated 1:1. This resampled dataset was then integrated with binary logistic regression analysis to construct a novel SMOTE‐based predictive model. The results demonstrated that the AUC of the P1 prediction model was lower than that of the P2 model in both the training and validation sets. Furthermore, the P2 model exhibited higher F‐score and PPV but a lower TPR compared with the P1 model. These findings suggest that the SMOTE‐based prediction model for AVF restenosis risk after PTA in MHD patients demonstrates satisfactory predictive accuracy. The calibration curve also indicated good agreement between predicted and observed outcomes, with well‐aligned calibration points. Additionally, the clinical decision curve derived from the nomogram revealed that employing this prediction model for timely, effective, and personalized intervention in high‐risk MHD patients could benefit the majority of this population, highlighting its considerable clinical utility.

Intradialytic hypotension was associated with higher restenosis risk in the SMOTE model. Whether preventing intradialytic hypotension reduces restenosis requires interventional studies. Multisegmental stenosis and buttonhole cannulation were associated with restenosis; these findings support careful surveillance and consideration of cannulation technique, but do not establish causality. Platelet count and CRP were independent risk factors in the original‐data model. Their roles in restenosis pathogenesis require further mechanistic and clinical validation. Serum albumin was significant only after SMOTE; therefore, no firm recommendation regarding high‐protein diet can be made from this study.

However, all samples were sourced from a single center, which may introduce selection bias, and the lack of external validation represents a limitation of the study. However, as this was a single‐center study, further multicenter research with larger datasets and external validation is warranted to confirm the generalizability of the model. The original training set had only 30 observed restenosis events for 11 predictors (EPV ≈ 2.7). Although SMOTE increased the minority class, synthetic samples do not provide new clinical information. The model therefore requires external validation with a larger real‐world cohort. The validation set contained only 15 restenosis events, which limits the stability of AUC and calibration estimates. Future studies should use a larger validation cohort or external validation.

## 5. Conclusion

In summary, multisegmental stenosis, cannulation method, platelet count, and CRP were independent risk factors for AVF restenosis within 12 months after PTA in MHD patients. The SMOTE‐based prediction model showed exploratory predictive accuracy (AUC = 0.868 in the validation set), but it requires external validation. Intradialytic hypotension and serum albumin became significant only after SMOTE and should not be regarded as independent risk factors without further validation.

## Author Contributions

Jianping Yang contributed to the study conception and design, analysis and interpretation of results, statistical processing, and manuscript drafting. Yamei Zhu participated in the data collection and manuscript revision. Xiaoqing Zhu and Chensi Dong were responsible for data collection and organization. Zhuangzhuang Guo performed statistical analyses. Mi Wang and Xueqiang Xu provided guidance on manuscript writing. Xianrong Xu oversaw quality control, review, and overall supervision of the study.

## Funding

This study was supported by the Nursing Program of Clinical Competence Enhancement Project 2022, JSPH‐NB‐2022‐13.

## Disclosure

All authors have read and approved the final paper.

## Ethics Statement

The study was conducted in accordance with the Declaration of Helsinki and was approved by the Institutional Ethics Committee of Jiangsu Provincial People′s Hospital (The First Affiliated Hospital of Nanjing Medical University, Jiangsu Provincial Maternity and Child Health Care Hospital) (Approval No. 2023‐SR‐136).

## Consent

Written informed consent was obtained from the participants prior to the study.

## Conflicts of Interest

The authors declare no conflicts of interest.

## Data Availability

The data that support the findings of this study are available from the corresponding author upon reasonable request.
